# Does metabolic water control the phosphate oxygen isotopes of microbial cells?

**DOI:** 10.3389/fmicb.2023.1277349

**Published:** 2023-11-20

**Authors:** Tal Weiner, Federica Tamburini, Nir Keren, Jonathan Keinan, Alon Angert

**Affiliations:** ^1^The Institute of Earth Sciences, The Hebrew University of Jerusalem, Jerusalem, Israel; ^2^Institute of Agricultural Science, ETH Zürich, Zurich, Switzerland; ^3^Department of Plant and Environmental Science, The Alexander Silberman Institute of Life Sciences, The Hebrew University of Jerusalem, Jerusalem, Israel

**Keywords:** metabolic water, oxygen stable isotopes, ambient water, carbon use efficiency, microbial cells

## Abstract

The oxygen isotopes ratio (δ^18^O) of microbial cell water strongly controls the δ^18^O of cell phosphate and of other oxygen-carrying moieties. Recently it was suggested that the isotopic ratio in cell water is controlled by metabolic water, which is the water produced by cellular respiration. This potentially has important implications for paleoclimate reconstruction, and for measuring microbial carbon use efficiency with the ^18^O-water method. Carbon use efficiency strongly controls soil organic matter preservation. Here, we directly tested the effect of metabolic water on microbial cells, by conducting experiments with varying the δ^18^O of headspace O_2_ and the medium water, and by measuring the δ^18^O of cell phosphate. The latter is usually assumed to be in isotopic equilibrium with the cell’s water. Our results showed no correlation between the δ^18^O of O_2_ and that of the cell phosphate, contradicting the hypothesis that metabolic water is an important driver of δ^18^O of microbial cell water. However, our labeled ^18^O water experiments indicated that only 43% of the oxygen in the cell’s phosphate is derived from equilibration with the medium water, during late-log to early-stationary growing phase. This could be explained by the isotopic effects of intra-and extra-cellular hydrolysis of organic compounds containing phosphate.

## Introduction

The oxygen isotope ratios in biominerals from hard tissues have been used extensively to reconstruct paleo-temperatures, paleo-environments, and the isotopic composition of the water in the environment. The studied hard tissues are usually composed of carbonates and phosphates and the measurements have been mainly of the isotopic ratio of ^18^O/^16^O (expressed in the delta notation as δ^18^O), but expanded recently also to ^17^O/^16^O (Tudge, 1960; Shackleton, 1967; Pearson, 2012; Passey and Levin, 2021). Other studies have focused on other chemical species, such as sulfates and nitrates, and the isotopic composition of oxygen in these species was used to reconstruct past environments and study ecosystem processes (Fritz et al., 1989; Casciotti et al., 2002). Implicit assumptions made for all these applications are that (1) the oxygen isotopic composition of intracellular water is transferred to oxygen-bearing chemical species, and (2) while intracellular water of large animals is isotopically different from ambient water, the intracellular water of small organisms (i.e., bacteria, plankton) and the ambient water exchange fast enough to be practically identical in terms of isotopic composition.

The assumption of isotopically identical ambient and intracellular water, however, might not be valid if the water inside the living cell is strongly controlled by metabolic water. Metabolic water is produced as a result of the aerobic cellular respiration process. In this process, oxygen atoms are transferred from O_2_ to H_2_O, following the canonical equation for respiration:


$$C_{6}H_{12}O_{6}+{\mathrm{6O}}_{2}\to {\mathrm{6CO}}_{2}+{\mathrm{6H}}_{2}O$$

Since the isotopic composition of atmospheric O_2_ is usually different from that of the water in the same environment, the contribution of metabolic water can change the intracellular water isotopic composition. The isotopic composition can then be carried over to the oxygen-bearing compounds of interest, like phosphates, carbonates and nitrates. The effect of metabolic water was recognized decades ago in the case of large land organisms like mammals, birds, and plants, which have limited exchange of cell water with the environment (Luz and Kolodny, 1985; Yakir, 1992; Sabat et al., 2021). However, for micro-organisms, the implicit assumption of fast exchange and negligible contribution of metabolic water was dominant until recently challenged with a series of experiments in which bacteria were grown in media with varying water oxygen isotopic compositions (Li et al., 2016). At the end of each experiment (during the late-log to early-stationary growing phase), the bacteria biomass was collected and the δ^18^O of phosphate (δ^18^O_P_) from DNA and from the total biomass (which includes inorganic cytosolic phosphate and organic-P) was measured. Based on the assumption that all the cell phosphate (i.e., cytosolic phosphate and phosphate bound to organic compounds) was in equilibrium with the cell water, this study concluded that ∼30% of the oxygen in DNA, and 40% of the total biomass phosphate of cells in this growing phase are derived from metabolic water. Based on this indirect finding, it was suggested that metabolic water strongly controls the isotopic composition of water in microbial cells.

The indirect evidence for the contribution of metabolic water to cell water was supported by previous research (Kreuzer-Martin et al., 2005), which measured the stable oxygen isotopes in water distilled from filtered microbial (*Escherichia coli*) “cell cakes.” These “cell cakes” contained both intercellular and medium water, and isotope labeling was used to distinguish between the two. That led to the conclusion that 70% of the intracellular water in log-phase *E. coli* cells was of metabolic origin.

Here, we directly tested the hypothesis that metabolic water has a significant effect on the cell water isotopic composition, and hence the total biomass δ^18^O_P_, by conducting experiments in which the bacteria were exposed not only to varying water isotopic composition, but also to varying headspace O_2_ isotopic composition. This approach was used in a recent study and proven successful to trace the incorporation of labeled O_2_ from the headspace to the metabolic water (Koch and Schwartz, 2023). The labeling of the headspace, which was not done in the previous phosphate studies, is expected to lead to changes in metabolic water oxygen isotopes ratios, and enables to test the hypothesis that the metabolic water signal has strong control over the cell water and hence the biomass δ^18^O_P_.

## Materials and methods

*Pseudomonas putida* was grown in a 1 L gas-tight Tedlar bag, equipped with a one-way Luer check-valve to release gas pressure buildup. Similarly to Li et al. (2016), the bacteria were grown in 200 mL LB medium composed of 2 g Tripton, 1 g yeast extract, 2 g NaCl and 0.2 mL 1 M NaOH, in either natural or δ^18^O enriched water (+108.5‰, all isotopic values are given vs. V-SMOW). An aliquot of the water used for the medium preparation was taken for oxygen stable isotopes analysis. A headspace of 800 mL gas was pre-introduced to the bag. The headspace was either air (δ^18^O of 23.9‰; Barkan and Luz, 2005) or a mixture of 632 mL N_2_ and 168 mL O_2_ a with a δ^18^O of −13.0‰. The bags with the medium and bacteria were shaken at 30°C for 17–21 h until late-log/early-stationary phase. At the end of the incubation, air from the headspace was sampled into glass flasks and measured for CO_2_ and O_2_ concentrations, in a system consisting of an infra-red gas analyzer (IRGA) for CO_2_ measurement and a fuel-cell based analyzer for measuring O_2_ (Hilman and Angert, 2016). This procedure was done in order to validate that the conditions remained aerobic and that O_2_ was consumed from the headspace.

The biomass was isolated through centrifugation (3,500 RPM for 15 min), rinsed with 0.03 M HEPES buffer (Sigma-Aldrich), and transferred to quartz vessels for UV radiation digestion. The digestion took place in DDW with 2–3 mL phosphate-free H_2_O_2_ (Sigma Aldrich) under UVC irradiation (18 lamps with a total power of 30 W). To evaluate the release of phosphate from the organic-P, one of the experiments was performed in duplicates, one in deionized water with δ^18^O of +0.2‰, and the second in water with δ^18^O of +97.1‰, the measured δ^18^O_P_ was 24.4‰ and 32.8‰ respectively. This enables us to calculate by an isotopic mass balance approach, the percentage of oxygen that was incorporated into the released phosphate from the water during the UV-induced mineralization (Liang and Blake, 2006). We used the following equations for calculation assuming *x* and *y* are equal in both samples:


(1) x×y+(1−x)×97.1=32.8

(2) x×y+(1−x)×0.2=24.4

Where *x* is the percentage of oxygen that originated from the biomass, 1 − *x* is the percentage of incorporated oxygen that originated from water, and y is the δ^18^O of the biomass. The percentage of incorporated oxygen that originated from water was found to be 9%, and the value of biomass δ^18^O_P_ was corrected accordingly in all other samples.

After the digestion of the biomass, the samples were kept refrigerated for 2 weeks. We found this stage useful for preventing precipitation of MoO_3_ during the next steps, which might be produced due to an excited state caused by the UVC radiation. The samples were acidified with 1 M HCl to prevent growth of microorganisms and were shaken with SuperliteTM DAX-8 resin (Sigma Aldrich) to remove any remaining organic matter.

The phosphate was purified according to Tamburini et al. (2010). Briefly, the phosphate was precipitated as ammonium phospho-molybdate (APM) using 35% NH_4_NO_3_ and 10% NH_4_Mo, vacuum filtered, rinsed with 5% NH_4_NO_3_, and finally dissolved in citric acid-NH_4_OH solution. The phosphate was re-precipitated as struvite using a magnesium-based reagent composed of MgCl_2_•6H_2_O, NH_4_Cl, and HCl, and 1:1 ammonia solution, vacuum filtered and rinsed with 1:20 ammonia solution and dissolved in 0.5 M HNO_3_. A cation resin AG50 × 8 resin (Bio-Rad) was used for removal of cations. The purification was followed by precipitation as silver phosphate using a mixture of 1.1 M AgNO_3_ and 2.72 M NH_4_OH, while KOH and HNO_3_ were added to adjust the pH (Mine et al., 2017). The precipitates were rinsed in water after centrifugation (3,500 RPM, 15 min) and were dried at 50°C. Phosphate concentrations were determined by colorimetry (Murphy and Riley, 1962).

The water samples were analyzed for δ^18^O by either headspace equilibration using Thermo Scientific GasBench II coupled to a Thermo Finnigan Delta Plus XL isotope-ratio mass spectrometer (at UC Davis), or by cavity ringdown spectroscopy using a Picarro L2140-i isotopic water analyzer (at the Hebrew University), described in detail by Keinan and Goldsmith (2023). In brief, liquid water samples were stored in 2 mL glass vials capped with blue polypropylene caps with red PTFE/white silicone septum and injected with a liquid autosampler (Picarro A0325) into a vaporizer module (Picarro A0211), using pure nitrogen (99.999%) as the carrier gas. Sample isotope ratios were standardized using a range of reference waters which have been calibrated against IAEA reference waters (VSMOW2, and SLAP2). For δ^18^O_P_ the Ag_3_PO_4_ was measured in a TC/EA (thermal conversion elemental analyzer, Vario Pyro Cube, Elementar GmbH, Germany) in pyrolysis mode, coupled in continuous flow with an IRMS (isotope ratio mass spectrometer, Isotprime, Elementar, GmbH, Germany). Two benzoic acid standards (IAEA 601: δ^18^O = 23.1‰, IAEA 602 δ^18^O = 71.3‰), an internal Ag_3_PO_4_ standard (Acros Organics, Geel, Belgium, δ^18^O = 14.2‰), and in-house standards were used for instrumental drift correction and isotopic calibration.

## Results and discussion

The headspace gases measurements showed considerable consumption of O_2_. The average O_2_ concentration in the incubation bags at the end of an experiment dropped from 20.95 to 7.5% ± 3.6%, while the average CO_2_ concentration increased from 0.04 to 7.8% ± 2.3% (Table 1). These concentration values correspond to a respiratory quotient (increase in CO_2_ divided by the decrease in O_2_) of 0.6 ± 0.1. These values indicate that aerobic respiration was dominant during the experiments despite the considerable oxygen consumption, since anaerobic respiration is associated with values above 1.0. Simple calculations showed the amount of O_2_ in the solution is small compared to the amount consumed from the headspace, since the medium contains less than 0.05 mmol of dissolved O_2_, while the headspace contains about 7 mmol of gaseous O_2_. Hence, the O_2_ consumed during the experiment is almost entirely derived from the headspace, and if metabolic water has a strong contribution to the cell water, the δ^18^O of the cell water should be affected by the δ^18^O of O_2_ in the headspace (which was varied between experiments).

**Table 1:** Results of headspace gases measurements at the end of each experiment.

Exp.	Exp. duration (h)	CO_2_ (%)	O_2_ (%)	RQ
1	19.91	4.0	14.3	0.59
2	19.91	5.8	11.6	0.62
3	19.91	5.6	11.3	0.58
4	19.91	4.9	12.5	0.57					
5	18.42	8.1	6.6	0.56
6	18.42	9.9	7.1	0.71
7	18.42	5.8	10.4	0.55
8	18.42	6.6	8.5	0.53					
9	18.92	6.8	6.0	0.45
10	18.92	7.5	7.0	0.54
11	18.92	7.1	7.5	0.53					
12	20.92	10.8	0.9	0.54
13	20.92	11.1	1.3	0.56
14	20.92	9.8	1.2	0.49
15	20.92	11.4	1.5	0.59					
16	17.08	1.8	14.3	0.26
17	17.08	9.5	6.1	0.64
18	17.08	8.8	5.6	0.57
19	17.08	0.4	18.5	0.14

RQ is the respiratory quotient (increase in CO_2_ divided by the decrease in O_2_, ΔCΟ2/−ΔΟ2).

The isotopic results are summarized in Table 2. To calculate the value of δ^18^O in phosphate in equilibrium with medium water, we used the equation from Chang and Blake (2015):


(3) $$1000^{*}\;\ln\;\alpha =14.43^{*}1000/T\left(K\right)-26.54$$

**Table 2:** Summary of all the isotopic data, grouped by experiments blocks.

Exp.	Headspace O_2_ δ^18^O	Medium water δ^18^O	δ^18^O_P_ measured	δ^18^O_P_ corrected for UV-introduced oxygen	Calculated equilibrium with medium water	Calculated equilibrium with 30% metabolic water
1	23.9	0.0	19.6	21.4	21.3	22.4
2	23.9	0.0	20.9	22.9	21.3	22.4
3	−13.0	0.0	21.1	23.0	21.3	11.4
4	−13.0	0.0	22.1	24.1	21.3	11.4							
5	23.9	11.9	21.0	22.0	33.2	30.8
6	23.9	11.9	19.1	20.9	33.2	30.8
7	−13.0	11.9	20.2	22.0	33.2	19.7
8	−13.0	11.9	22.9	25.0	33.2	19.7							
9	−13.0	0.3	19.2	21.1	21.6	11.6
10	23.9	0.3	19.5	21.1	21.6	22.7
11	23.9	0.3	19.3	21.3	21.6	22.7							
12	23.8	3.3	26.1	28.3	24.5	24.7
13	23.8	3.3	23.1	25.1	24.5	24.7
14	−13.0	3.3	25.3	27.4	24.5	13.7
15	−13.0	3.3	27.2	29.5	24.5	13.7							
16	23.8	87.2	50.7	55.4	108.5	83.5
17	23.8	87.2	56.1	61.5	108.5	83.5
18	−13.0	87.2	53.6	58.6	108.5	72.4
19	−13.0	87.2	59.1	64.7	108.5	72.4

All isotopic values are in delta permil vs. V-SMOW. The experiments were performed with both DDW water at natural abundance and ^18^O-labeled DDW water, and both with air headspace and light-O_2_ headspace. The value of “δ^18^OP corrected for UV introduced oxygen,” is the measured value with correction for 9% contribution from water oxygen incorporation during the UV treatment (see methods). See text for explanations of equilibrium calculations. Note that the light-O_2_ headspace do not cause lighter δ^18^OP, in contrast to the calculated equilibrium value when assuming 30% contribution of metabolic water.

And then used the relationship:


(4) $$\delta^{18}O_{P}\left(\mathrm{equilibrium}\right)=\delta^{18}O_{\mathrm{medium water}}+1000^{*}\ln\;\alpha$$

To check for possible effect of metabolic water, we calculate the equilibrium not just with the medium water, but also with assumed cell water composed of 70% medium water and 30% metabolic water (Table 2), as asserted in Li et al. (2016). The δ^18^O of metabolic water was calculated based on the δ^18^O value of the O_2_ in the bag headspace, and the known −20‰ fractionation of the consumed O_2_ during aerobic respiration (Guy et al., 1989; Bender et al., 1994). It should be noted that this is a simple approach, which ignore possible effect of fractionation in the gas-water interface and the effect of slow diffusion through this interface, as well as the consumption effect on the δ^18^O of the remaining O_2_. However, these effects are not related to the δ^18^O of the O_2_ in the headspace, and hence, the calculated difference between the metabolic water under different headspace O_2_ is a good estimate.

**Figure 1:**
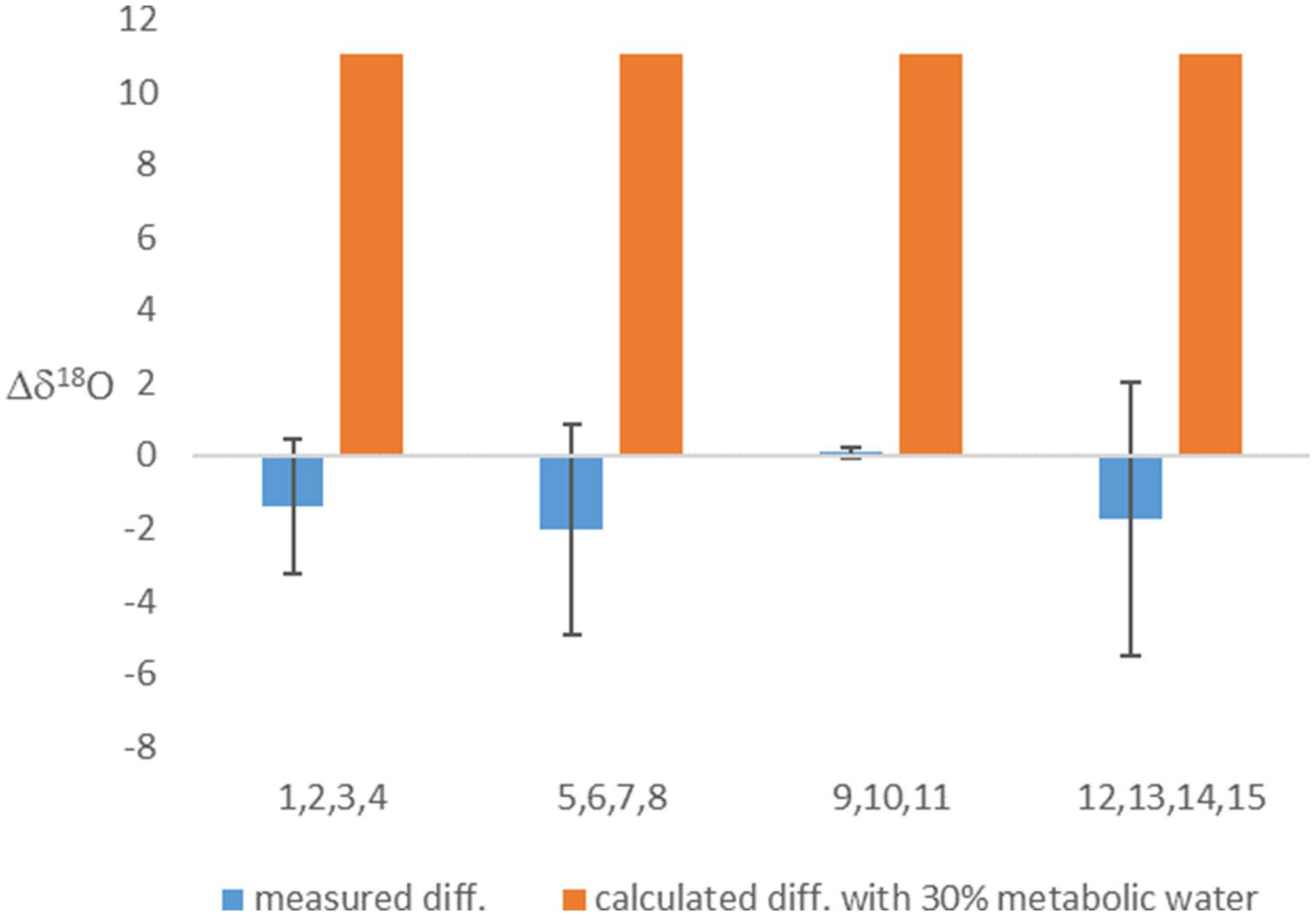
The difference between the δ^18^O_P_ of experiments with headspace of air-O_2_ and light O_2_. Presented are the measured values, and calculated values based on the assumption that 30% of cell water is metabolic water. Averages (based on experiments numbers indicated in the x-axis and grouped in blocks) and error bars are shown.

Considering metabolic water made the calculated equilibrated δ^18^O_P_ lighter (Table 2). This effect on the calculation was weaker in the experiment in which the headspace was air (with δ^18^O of O_2_ of 23.9‰), but, as expected, had a marked effect when light-O_2_ (δ^18^O of −13.0‰) was used in the headspace. This calculation shows that if metabolic water was indeed contributing 30% of the internal cell water, the δ^18^O_P_ should have been 11‰ lighter in the experiments in which light-O_2_ was in the headspace than in the experiments with atmospheric air. However, this is not the case (Table 2; Figure 1). This result suggests that fast exchange between the cell and the ambient water might have erased the contribution of metabolic water to the cell water.

**Figure 2:**
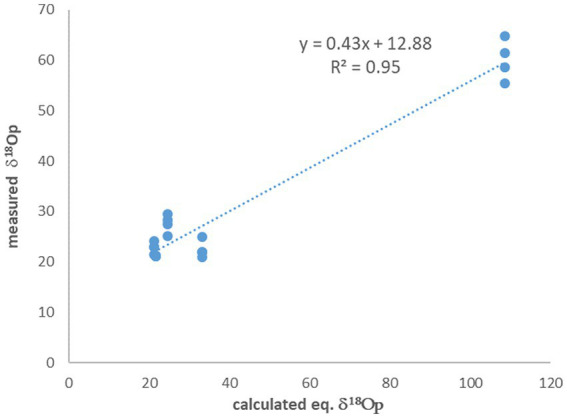
The measured δ^18^O_P_ vs. the calculated δ^18^O_P_ in equilibrium with ambient water. The slope indicate that only 43% of the phosphate oxygen is derived from the water.

The experiments with no ^18^O-labeled water additions to the medium show δ^18^O_P_ values which are close to equilibrium with the medium water. On average, the difference between the calculated equilibrium between phosphate and medium water and the measured value (after correction for oxygen introduced during UV treatment) is only 2.7‰, but with quite a large standard deviation of 2.4‰. However, for the experiments in which ^18^O-labeled water was added to the medium and enriched it to 87.2‰, this difference is one order of magnitude larger (Table 2) and is 48.5% ± 4.0‰. A known approach to estimate the control of ambient water on the phosphate oxygen isotopes, is to plot the measured δ^18^O_P_ versus the calculated δ^18^O_P_ in equilibrium with ambient water (Li et al., 2016). In our experiments the fitted linear slope on this plot is 0.43 (Figure 2), indicating that only 43% of the oxygen atoms in the cell phosphate originate from the medium water, while the rest must have another source.

The results from the experiments with ^18^O-labeled water in the medium are in general agreement with (Li et al., 2016), who reported that ∼40% of the O in biomass PO_4_ is not derived from extracellular water. In their paper, they suggested that the gap between the measured values and the values calculated by assuming equilibrium with medium water, could be explained if 40% of the biomass phosphate oxygen came from metabolic water with δ^18^O = −3.5‰ (Li et al., 2016). We can calculate the expected value for metabolic water from the measured δ^18^O of atmospheric O_2_, which is 23.9‰ (Barkan and Luz, 2005), and the known isotopic fractionation during respiration which is ~−20‰. This yields an estimate of ~3.9‰ (23.9–20), which is ~7‰ heavier than the value of −3.5‰ estimated by Li et al. (2016). The additional isotopic effects on the metabolic water, discussed above, will only increase this mismatch. Thus, this mismatch strengthens our conclusion that metabolic water is not a significant contributor to cell water, and that the deviations from the calculated equilibrium with ambient water are due to other reasons.

**Figure 3:**
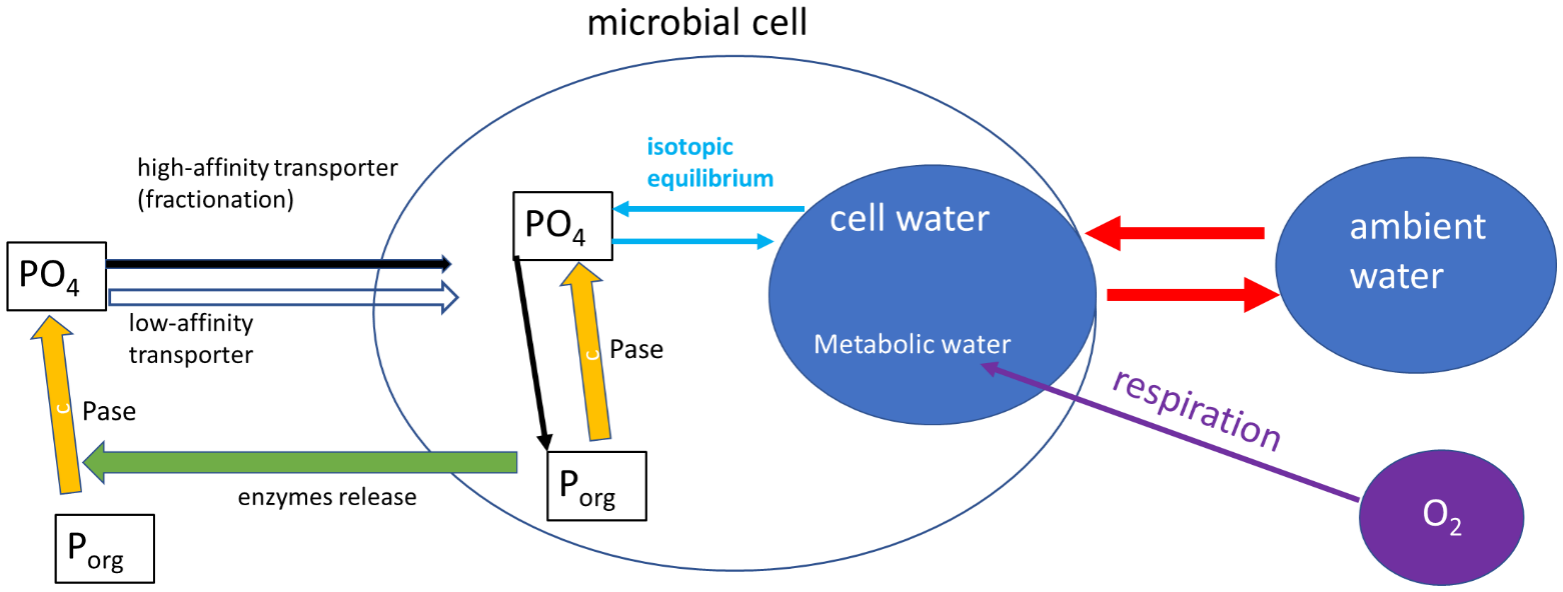
Schematic representation (after Pistocchi et al., 2020) of the reactions affecting phosphate oxygen isotopes in a microbial cell. This included inherence from organic-P sources, fractionation by phosphatases (Pase) and isotopic equilibrium with cell water. The cell water is controlled by exchange with ambient water which is fast, and a slow contribution of metabolic water.

What can drive this deviation from the expected equilibrium? One major assumption of most studies dealing with intracellular ^18^O in phosphate is that the oxygen isotopes in the cell P (biomass plus cytosolic phosphate) are only controlled by equilibrium with the cell-water. This ignores many intracellular reactions (e.g., ATP driven energy conversion reactions, organic P synthesis by phosphotransferases, phosphorylation and dephosphorylation of molecules and proteins), which are known to have possible isotope effects (see Hengge, 2002 and refs therein), but are not yet characterized for their isotopic fractionation. One possible explanation for the deviation from equilibrium observed in our experiments could be the activity of other enzymatic reactions (Figure 3). Indeed, phosphate released from the cleavage of organic P is known to be affected by enzyme-specific kinetic fractionation, on top of the carried inherent signature of the phosphate from the organic molecule (Liang and Blake, 2006; Liang and Blake, 2009; von Sperber et al., 2014). The medium in which the bacteria were grown during our experiments (and also in Li et al., 2016) included yeast extract, which is a complex nutrient-rich source and contains about 2.5% of organic P (Dzurendova et al., 2020). Previous research (Pistocchi et al., 2020) also found that in some soils the oxygen isotopic ratios in microbial P (mainly cytosolic) can be far from equilibrium and attributed this to the intracellular enzymatic release of phosphate from organic-P. In addition, phosphate uptake by microbial cells high-efficiency transporter was shown to involve isotopic fractionation (Lis et al., 2019). It has to be noted that both the current and the previously cited works targeting metabolic water and ^18^O in phosphate, targeted the sum of cytosolic and organic (UV-degradable) phosphate. Different results might be expected if these phosphate pools could be separately analyzed.

These results are important not only for paleoclimate reconstructions, but also for measurements of carbon turnover in soils, which strongly controls atmospheric CO_2_ and hence climate. Recent modeling efforts showed that the amount of carbon stored in soils is highly sensitive to carbon use efficiency (CUE) which is the ratio between the carbon used for microbial growth and the carbon emitted in microbial respiration (Manzoni et al., 2012; Tao et al., 2023). The CUE is often measured by labeling the soil water with ^18^O, and tracing this label incorporation into the microbial DNA. While our results showed that metabolic water production do not dilute this label (in agreement with Wang et al., 2022), the incorporation to the DNA of phosphate which inherent it oxygen atoms from organic-P, will invalidate this method assumptions (Pold et al., 2020). However, it should be noted that the DNA δ^18^O is also controlled by the oxygen atoms in the deoxyribose sugars and in the DNA bases, with unknown direct contribution of ambient water to this oxygen. Hence, the fraction of oxygen derived from ambient water in total DNA should be tested in future studies, because of its implication for CUE measurements.

In conclusion, our experiments directly show that the deviation from isotopic equilibrium between the ambient water and the oxygen in phosphate is not derived from the contribution of metabolic water to the cell water. We suggest that this deviation is most likely the result of intracellular enzyme activity, which is associated with fractionation and inheritance isotopes effects. However, more research is needed to clarify the various processes which control the phosphate oxygen isotopes in microbial cells.

## Data availability statement

The original contributions presented in the study are included in the article/supplementary material, further inquiries can be directed to the corresponding author.

## Author contributions

TW: Writing – original draft, Writing – review & editing, Data curation, Investigation. FT: Writing – original draft, Writing – review & editing, Conceptualization. NK: Writing – review & editing, Resources. JK: Resources, Writing – review & editing. AA: Writing – review & editing, Conceptualization, Funding acquisition, Project administration, Supervision, Visualization, Writing – original draft.
